# Higher IGFBP-1 to IGF-1 serum ratio predicts unfavourable survival in patients with nasopharyngeal carcinoma

**DOI:** 10.1186/s12885-017-3068-0

**Published:** 2017-01-31

**Authors:** Xinwei Feng, Jianhua Lin, Shan Xing, Wanli Liu, Ge Zhang

**Affiliations:** 1 Department of Microbial and Biochemical Pharmacy, School of Pharmaceutical Sciences, Sun Yat-sen University, No.132 Waihuandong Road, University Town, Guangzhou, 510006 China; 2 Department of Clinical Laboratory Medicine, Sun Yat-sen University Cancer Center, Guangzhou, China

**Keywords:** Insulin-like growth factor binding protein 1, Insulin-like growth factor 1, Nasopharyngeal carcinoma, Clinical prognosis

## Abstract

**Background:**

The insulin-like growth factor (IGF) system plays an important role in the development and progression of cancer. However, little is known about the expression of the IGF system components and their clinicopathological significance and prognostic value in nasopharyngeal carcinoma (NPC).

**Methods:**

IGF system components (IGF-1, IGF-2, IGF-1SR, IGFBP-1, IGFBP-2, IGFBP-3, IGFBP-4 and IGFBP-6) were quantified from the plasma of NPC patients and healthy individuals using the RayBio Human Cytokine Antibody Array. IGFBP-1 and IGF-1 mRNA levels were quantified by real-time qPCR, and protein expression was detected by western blot in nine NPC cell lines and four immortalized nasopharyngeal epithelial (NPE) cell lines. Tissue-specific expression of IGFBP-1 and IGF-1 was detected by immunohistochemistry in paraffin-embedded NPC tissues. ELISA analysis was used to measure the serum levels of IGFBP-1 and IGF-1 in 142 NPC patients and 128 healthy controls and determine potential correlation with clinicopathological parameters.

**Results:**

Significantly higher levels of circulating IGFBP-1 and lower levels of IGF-1 and IGF-2 were detected in NPC patients compared to healthy controls by Cytokine Antibody Array analyses (*P =* 0.034*,* 0.012, 0.046*,* respectively). IGFBP-1 expression was detected in the majority of NPC cell lines, but not in NPE cell lines, and was shown to localize to the nucleus of tumour cells, in contrast to the cytoplasmic staining observed in normal cells. Importantly, IGFBP-1 expression was stronger in NPC tumour tissues compared to peritumoural tissues. In contrast, IGF-1 expression was weak or absent in NPC and NPE cell lines, with the exception of the EBV-infected C666 cell line, and was found to be expressed at lower levels in tumour tissues compared to tumour-adjacent normal tissue. Levels of serum IGFBP-1 were shown to be significantly higher in patients with NPCs compared to healthy control individuals (55.23 ± 41.25 μg/L *vs*. 32.08 ± 29.73 μg/L, *P* < 0.001), whereas serum levels of IGF-1 were significantly lower in NPC patients compared to healthy controls (98.14 ± 71.48 μg/L *vs*. 164.01 ± 92.08 μg/L, *P* = 0.001). Consistently, the IGFBP-1/IGF-1 serum ratio was shown to be significantly higher in NPC patients compared to healthy control individuals (*P* = 0.002). Serum levels of IGFBP-1 and the IGFBP-1/IGF-1 ratio significantly correlated with age (*P* = 0.020*; P* = 0.016), WHO histological classification (*P* = 0.044; *P* = 0.048), titre of EA (EB Virus Capsid Antigen-IgA) and NPC (*P* = 0.015*; P* = 0.016). In contrast, higher IGFBP-1 serum levels and IGFBP-1/IGF-1 ratio significantly correlated with poor RFS (*P* = 0.046*; P* = 0.037) and OS (*P* = 0.038*; P* = 0.009). Multivariate analysis revealed that the IGFBP-1/IGF-1 ratio, but not serum IGFBP-1 level, represents an independent risk factor for poor RFS (*P* = 0.044) and OS (*P* = 0.035).

**Conclusions:**

A higher IGFBP-1/IGF-1 serum ratio is significantly associated with poor prognosis in NPC patients.

## Background

Nasopharyngeal carcinoma (NPC) is a malignant head and neck tumour with a distinct racial and geographical distribution that is highly prevalent in Southeast Asia [1]. Although radiotherapy and chemotherapy are efficient therapeutic approaches for treating NPC, the disease remains a deadly due to late presentation of the disease and poor prognosis. Because NPC tumours are asymptomatic, advanced disease at time of diagnosis and high rates of recurrence and metastasis underlie the high mortality rate in NPC patients. Therefore, finding new biomarkers or risk factors will contribute to earlier diagnosis and better prognosis for NPC patients.

The insulin-like growth factor (IGF) system consists of a complex network of ligands (IGF-1 and 2), their cognate receptors (IGFR-1 and 2), IGF-binding proteins (IGFBP1-6), and IGFBP proteases. The IGF signalling pathway, which facilitates communication between cells and their physiologic environment, may be involved in human cancer progression and can be targeted for therapeutic intervention [2]. Within the blood stream, IGF-1 is bound to IGFBPs and activates IGF-1R following its release from the complex. The interaction between IGFs, IGFBPs and IGFRs promotes cellular proliferation and inhibits apoptosis [3].

Several studies have reported a correlation between circulating levels of IGF-1 and IGFBP-1 in healthy people and the risk of cancer development. IGF-1 plasma or serum levels have been reported to be increased in patients with a variety of cancers, including colorectal adenoma, malignant melanoma, breast cancer, non-small cell lung cancer [4–6]. However, conflicting results have been observed in studies conducted in prostate cancer, epithelial ovarian cancer, breast cancer, and oral cancer [7–10]. In addition, serum levels of IGFBP-1 have been reported to be increased in metastatic prostate and oral cancers, but not in pancreatic, non-metastatic colorectal or endometrial cancers [11–13]. Despite considerable research, the role of IGFs in cancer remains unclear, and clinical trials have been unsuccessful [14]. Moreover, how IGF-1 and IGFBP-1 are regulated at the expression level remains equivocal in tumour tissues and within the circulating blood stream.

In the present study, we examined the expression patterns of IGF-1 and IGFBP-1 in different NPC and normal nasopharyngeal epithelial (NPE) cells lines. Furthermore, we assessed the serum levels of IGF-1 and IGFBP-1 in NPC patients and healthy control individuals and determined whether altered IGF-1 and IGFBP-1 levels were associated with clinical outcome to assess the potential value of IGF-1 or IGFBP-1 as a prognostic biomarker for NPC.

## Methods

### Patients, blood and tissue samples

Plasma from 10 NPC patients and 10 healthy volunteers were obtained in October 2012 and used for 8 IGF-related cytokine arrays. Plasma samples were stored at 80 °C and were measured in 3 months.

Sera used for IGF-1 and IGFBP-1 arrays were obtained from 143 patients with NPC between November 2005 and October 2008. The cohort consisted of 119 male patients and 24 female patients. Patients ranged in age from 15 to 71 years (mean, 49.6 years). All sera and plasma were collected from NPC patients at the time of diagnosis and prior to tumour radiation therapy or surgery. The 143 patient characteristics are described in Table 1.

**Table 1:** Clinical characteristic of 142 patients with NPC

Characteristic	No. (%)
Sex	
Male	118 (83)
Female	24 (17)
AGE	
Median	49.6
Range	15–71
≤ 45	73 (51)
> 45	69 (49)
Follow-up Time	
Median (range)	73 (13–125)
Tumor Size	
T1 + T2	27 (19)
T3 + T4	115 (81)
Lymphoid nodal states	
N0-1	76 (53)
N2-3	66 (47)
Clinical Stage	
1 + 2	15 (11)
3 + 4	127 (89)
Local-regional relapse	
Yes	16 (11)
No	126 (89)
Metastasis	
Yes	4 (3)
No	138 (97)
WHO histological classification	
NKUC	131 (92)
NKDC	11 (8)
OS rate (%)	
5-year	78.2%

Overall survival (OS) was defined as the interval between the date of surgery and the date of death or the last known follow-up visit. Relapse-free survival (RFS) was defined as the interval between the operation and the date that tumour recurrence or metastasis was diagnosed. All follow-up data from the NPC patients used in this study were available and complete. A total of 31 (21.8%) patients died during follow-up period.

Sera from 128 healthy volunteers (95 males, 33 females) with ages ranging from 21 to 77 years (mean: 47.8 years) were collected and used as controls. Healthy controls were selected from an archive of blood samples, and the control samples were matched as closely as possible to the NPC group with respect to previous handling and the time period of sample collection.

A 5-ml blood sample from each participant was allowed to clot for 30 to 60 min at room temperature. Each clotted sample was centrifuged at 1500 g for 10 min. All sera were then aliquoted and frozen at −80 °C until use.

Paraffin-embedded tumour tissue samples were obtained from 20 NPC patients who underwent surgery between May and December of 2013. None of the patients had received anticancer treatment prior to surgery, and all of the patients had histologically confirmed primary NPC in this retrospective study.

All of the blood and tissues sample were collected at the Cancer Center of Sun Yat-sen University. The study was approved by the Ethics Committee of Sun Yat-sen University Cancer Center and informed consent was obtained from each patient.

### Cytokine array

Eight IGF system members (IGF-1, IGF-2, IGF-1SR, IGFBP-1, IGFBP-2, IGFBP-3, IGFBP-4 and IGFBP-6) were quantified in the plasma of 10 NPC patients and healthy volunteers using the RayBio Human Cytokine Antibody Array (#AAH-CYT-5, RayBiotech Inc, GA, USA) according to the manufacturer’s instruction.

### Cell culture

Two well-differentiated NPC cell lines CNE1 and HK1, three poorly-differentiated NPC cell lines (CNE2, HONE1, SUNE1) and two SUNE-1 subclones (6–10B and 5–8 F) were cultured in DMEM medium (Sigma, Saint Louis, MO, USA). Two undifferentiated NPC cell lines, C666-1 (EBV-positive) and SUNE2 (extremely low concentrations of EBV), were cultured in RPMI 1640 medium (Sigma). Immortalized nasopharyngeal epithelial (NPE) cell lines (N5-Tert, N5Bmi-1, N2-Bmi-1) and normal NPE cell lines (NP460) were cultured in Keratinocyte-SFM medium (10744–019, Gibco) and used as a control. All of the cell lines were maintained in our laboratory, and all media were supplemented with 10% foetal bovine serum (Sigma).

### RNA preparation and quantitative real-time PCR

Total RNA was extracted from NPC and nasopharyngeal epithelial cells lines using the Trizol reagent (Invitrogen, USA) according to the manufacturer’s instruction. Reverse transcription of total RNA (2 μg) was performed using SuperScript II reverse transcriptase (GIBCO BRL, Grand Island, NY, USA). The quantification of target and reference glyceraldehyde-3-phosphate dehydrogenase (GAPDH) genes was performed using the Power SYBR Green qPCR SuperMix-UDG (Invitrogen, USA) on an iCycler (Bio-Rad, Hercules, CA, USA). CT is defined as the cycle at which the fluorescence is determined to be statistically significant above background. The relative mRNA expression was normalized to the expression of GAPDH, which yielded a 2^-ΔCT^ value (ΔCT = CT_(target gene)_–CT_(GAPDH)_). All reactions were performed in triplicate in three independent experiments. The primers used for real-time RT-PCR were as follows: IGF-1: forward 5′- GCT CTT CAG TTC GTG TGT GGA −3′ and reverse 5′- GCC TCC TTA GAT CAC AGC TCC −3′; IGFBP-1: forward 5′-CTA TGA TGG CTC GAA GGC TC-3 ′ and reverse 5′-CCC ATT CCA AGG GTA GAC G-3′; GAPDH: forward 5′-GCA CCG TCA AGG CTG AG AAC-3′ and reverse 5 ′-TGG TGA AGA CGC CAG TGG A-3′.

### Western blot

Total protein was extracted using a lysis buffer and protease inhibitor (Beyotime Biotechnology, China). Equivalent protein amounts were denatured in an SDS sample buffer and then were separated by SDS-PAGE and transferred onto polyvinylidene difluoride membrane. After being blocked with 5% non-fat dry milk in PBS containing 0.05% Tween-20, the blotted membranes were incubated with anti-human IGF-1 and IGFBP-1 antibodies, (1:5000, 1:1000, respectively, R&D systems, USA) and then incubated with a secondary antibody (1:5000, Boster, China). GAPDH protein levels were also determined by using the specific antibody (1:1000, Boster, China) as a loading control.

### Immunohistochemistry

Formalin-fixed, paraffin-embedded NPC sections were incubated with a goat polyclonal anti-IGF-1 (1:100, AF-291-NA, R&D, USA) or anti-IGFBP-1 antibody (1:100, AF871, R&D, USA) overnight at 4 °C. After washing in PBST, the tissue sections were treated with a horseradish peroxidase-conjugated anti-goat secondary antibody (1:1000, Zymed). The tissue sections were then developed with 3-diaminobenzidine tetrahydrochloride for 10 s, followed by counterstaining with 10% Mayer’s haematoxylin. The degree of staining was reviewed by two independent observers.

### ELISA

Serum IGF-1 and IGFBP-1 levels were determined by double-antibody sandwich ELISA according to the manufacturer’s instructions (DY291, DY871, R&D systems, USA). Briefly, 96-well microplates were coated with 100 μl/well of the capture antibody (mouse anti-human IGF-1 or IGFBP-1, 4.0 μg/ml) overnight at 4 °C. After blocking with 3% BSA, 100 μl of the test samples (1:100 diluted in 1% BSA) was added and incubated for 2 h at room temperature. Subsequently, 100 μl/well of the detection antibody (biotinylated goat anti-human IGF-1 (150 ng/ml) or IGFBP-1(400 ng/ml)) was added and incubated for 2 h at room temperature. Next, 100 μl/well of Streptavidin-HRP (1:200) was added and incubated for 20 min at room temperature. Finally, the substrate (tetramethylbenzidine) solution was added, and the reaction was terminated using 2 N H_2_SO_4_ and read at an OD of 450 nm. Each test included a standard control (CV = 12%).

### Statistical analysis

All statistical analyses were carried out using the SPSS 20.0 statistical software package (SPSS Inc., Chicago, IL). The Mann–Whitney *U* test was used to evaluate the difference in IGF-1 and IGFBP-1 serum levels between NPC patients and healthy controls. Pearson's chi-squared test was used to analyse the association between IGF-1 and IGFBP-1 levels and the observed clinicopathological characteristics of patients with NPC. Survival curves were plotted by the Kaplan-Meier method and compared using the log rank test. The significance of various variables for survival was analysed using the Cox proportional hazards model (univariate and multivariate analysis). *P* < 0.05 was considered to be statistically significant in all cases.

## Results

### Circulating levels of IGF-related cytokines differ between NPC patients and healthy control individuals

**Fig. 1:**
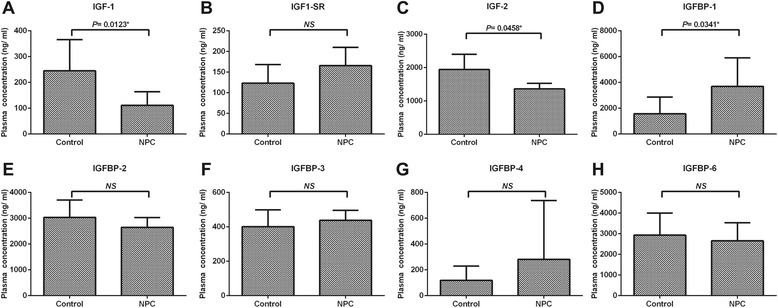
Characterization of plasma IGF-related cytokines levels in NPC patients and control individuals. Plasma levels of the following cytokines were analysed in NPC patient and control sera: **a** IGF-1; **b** IGF1-sR; **c** IGF-2 **d** IGFBP-1; **e** IGFBP-2; **f** IGFBP-3; **g** IGFBP-4; **h** IGFBP-6

The plasma level of eight members of the IGF system, including IGF ligands (IGF-1 and IGF-2), IGF-1 soluble receptor (IGF-1 sR), and IGF binding protein (IGFBP-1, IGFBP-2, IGFBP-3, IGFBP-4, and IGFBP-6) were detected in healthy controls and patients with NPC (*n* = 10, respectively) using a Human Cytokine Antibody Array (Fig. 1). The levels of IGF-1 and IGF-2 from NPC patients were significantly lower compared to healthy control individuals (*P* = 0.012, *P* = 0.046, respectively). Interestingly, while the plasma level of IGFBP-1 from NPC patients was significantly higher than in healthy control volunteers (*P* = 0.034), no significant differences were observed between the plasma levels of the other IGF soluble receptor or binding proteins, including IGF-1SR, IGFBP-2, IGFBP-3, IGFBP-4 or IGFBP-6. Fourthermore, serum level of IGF-1, IGF-2 and IGFBP-1 were analyzed by ELISA in preliminary experiments (*n* = 24), there was no significant difference in serum IGF2 level between NPC patients and health group. Together, these results suggest that NPC patients display increased circulating levels of IGFBP-1 and decreased levels of IGF-1.

### Characterization of IGFBP-1 and IGF-1 expression in NPC and NPE cell lines

**Fig. 2:**
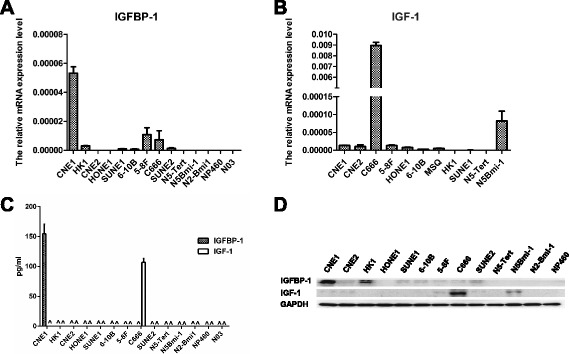
Expression of IGFBP-1 and IGF-1 in NPC and immortalized nasopharyngeal epithelial cell lines. RNA or protein was harvested from NPC and immortalized nasopharyngeal epithelial cell lines and characterized for mRNA expression of **a** IGFBP-1 and **b** IGF-1, and protein expression of IGFBP-1 and IGF-1 in culture medium **c** or cells **d**

Real-time PCR and western blot analysis showed that IGFBP-1 and IGF-1 were differentially expressed at both the mRNA and protein level in 9 NPC cell lines (CNE1, CNE2, HONE1, HK1, SUNE1, 6–10B and 5–8 F, C666 and SUNE2) and 4 NPE cell lines (N5-Tert, N5-Bmi-1,N2-Bmi-1 and NP460) (Fig. 2a-c). IGFBP-1 mRNA and protein were expressed in most of the NPC cell lines, with the exception of HONE1. CNE1 cells had the highest level of IGFBP-1, and moderate or weaker levels of expression were observed in the other 6 NPC cell lines. However, IGFBP-1 expression was not detected in any of the four NPE cell lines. In addition, ELISA analysis of cell supernatant showed that the secretory level of IGFBP-1 was below the detection limit in most the cell lines, with the exception of CEN1 (Fig. 2c).

IGF-1 mRNA and protein were detected in only 5 NPC cell lines, with the highest expression level of IGF-1 observed in C666 cells, which consistently harbours Epstein-Barr virus (EBV), and weaker expression of IGF-1 detected in three poorly differentiated NPC cell lines (CNE2, 5-F8, SUNE2) and well-differentiated cell lines CNE1. IGF-1 expression was shown to be absent in the other 4 NPC cell lines. Moreover, mRNA and protein levels of IGF-1 were expressed at moderate levels in the immortalized NPE cell line, N5Bmi-1, but not in the other 3 NPE cell lines. ELISA analysis showed that detectable secretory IGF-1 only in C666 cell line.

Those results showed that the stronger expression of IGFBP-1 in tumour cell lines than the normal cell lines, but weak expression of IGF-1 in both of tumour and normal cell lines.

### Characterization of IGFBP-1 and IGF-1 expression in NPC tissues

**Fig. 3:**
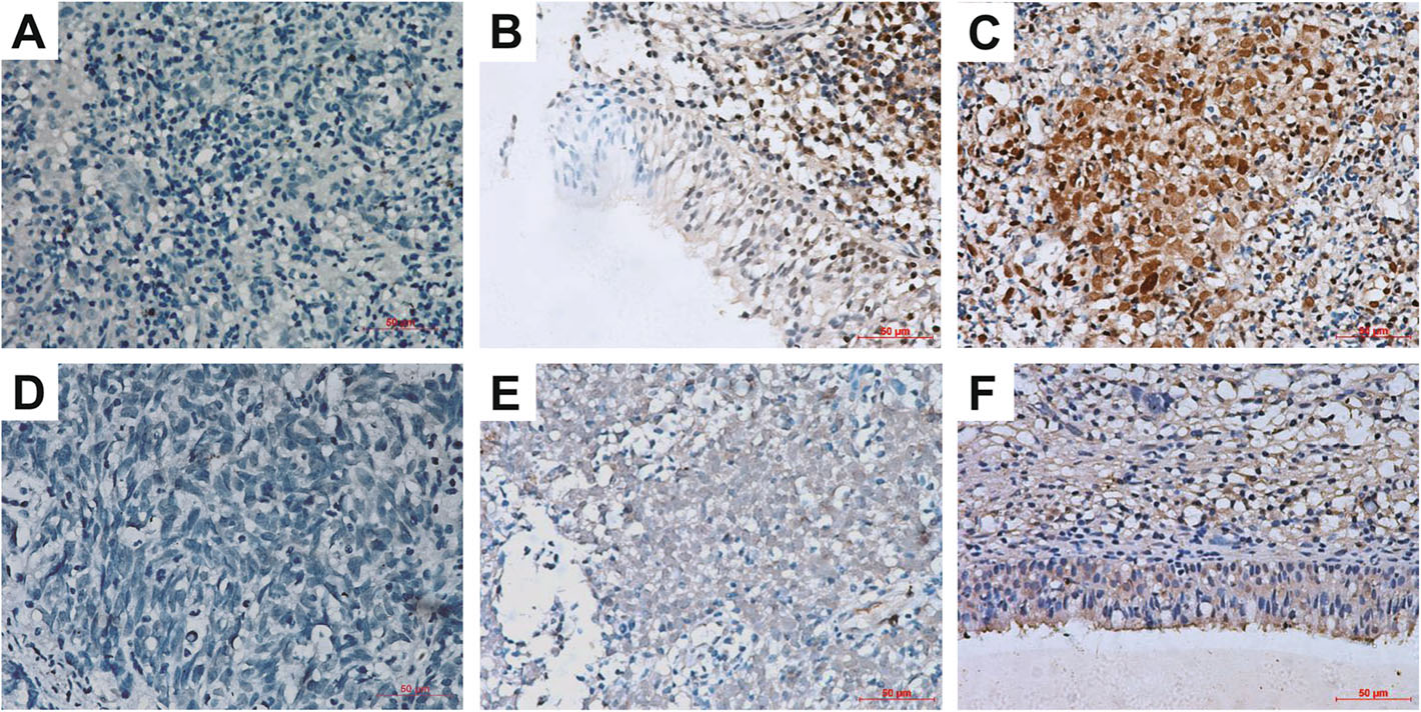
Expression analysis of IGFBP-1 and IGF-1 in NPC by immunohistochemistry. NPC slides were process for immunohistochemistry of IGFBP-1 and IGF-1 and the following observations were made: **a**-**g**. (×200). **a** Negative stain of IGFBP-1 in NPC tissues. **b** Low expression level of IGFBP-1 in adjacent non-tumourous tissue. **c** High expression level of IGFBP-1 in NPC tissues. **d** Negative stain of IGF-1 in NPC tissues. **e** Low expression of IGF-1 in NPC tissues. **f** High expression of IGF-1 in adjacent non-tumourous tissue. Bar, 50 μm

We examined the expression of IGFBP-1 and IGF-1 in 16 paraffin-embedded archived NPC tissues by immunocytochemistry. IGFBP-1 protein was detected in all of the 16 NPC tumour tissues (100%) and in 3 of the 16 normal adjacent tissues (18.75%). Immunostaining of IGFBP-1 was mainly detected in the nuclei of cancer cells and cytoplasm of normal cells, with stronger IGFBP-1 immunostaining present in tumour tissues compared to peritumoural tissues (Fig. 3a-c). In addition, IGF-1 protein was detected in 6 of the 16 NPC tumour tissues (37.5%) and in 14 of the 16 tumour-adjacent normal tissues (87.5%) with varied IGF-1 immunoreactivity. IGF-1 was shown localize to the cytoplasm of malignant tumour cells and surrounding stromal cells, while stronger cytoplasmic staining of IGF-1 was observed in the normal epithelial and stromal cells of adjacent non-tumourous tissue (Fig. 3d-f). Together, these results illustrate that IGFBP-1 is strongly expressed in tumour tissue, while IGF-1 expression is elevated in normal tissue.

### Serum levels of IGFBP-1 and IGF-1 correlate with distinct NPC clinicopathological characteristics

**Fig. 4:**
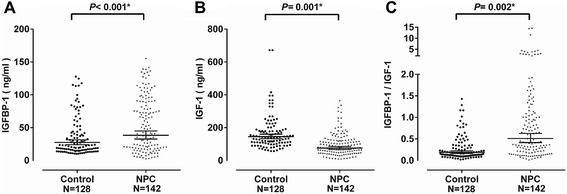
Comparison of serum levels of IGFBP-1, IGF-1 and r IGFBP-1/IGF-1 in NPC and control groups. Serum levels were quantified from NPC and control patients of **a** serum IGFBP-1 level, **b** serum IGF-1 level and **c** Ratio of serum IGFBP-1 to IGF-1

The serum levels of IGFBP-1 and IGF-1 were measured in healthy control individuals (*n* = 128) and NPC patients (*n* = 142), respectively (Fig. 4). The mean concentration of serum IGFBP-1 levels in NPC cases (55.23 ± 41.25 μg/L) was significantly higher compared to control cases (32.08 ± 29.73 μg/L, *P* < 0.001) (Fig. 4a). In contrast, the mean concentration of serum IGF-1 levels in NPC cases (98.14 ± 71.48 μg/L) was significantly lower compared to control cases (164.01 ± 92.08 μg/L, *P* = 0.01) (Fig. 4b). The ratio of IGFBP-1/IGF-1 in NPC patient serum was shown to be significantly higher compared to control volunteers (*P* = 0.002) (Fig. 4c).

Next, we assessed the potential correlation between serum levels of IGFBP-1, IGF-1 and clinical parameters, including tumour node metastasis (TNM) stage, tumour size, lymphoid nodal states, clinical stage, etc. The level of serum IGFBP-1 significantly correlated with age (*P* = 0.020), WHO histological classification (*P* = 0.044) and titre of EA (EBV early antigen-IgA) of NPC (*P* = 0.015). In addition, IGF-1 serum levels were shown to significantly correlate with gender, but not with other clinical parameters, and the mean level of IGF-1 in female patients was higher than in males (*P* = 0.018). Furthermore, we found that the IGFBP-1/IGF-1 ratio significantly correlated with age (*P* = 0.016), WHO histological classification (*P* = 0.048) and titre of EA of NPC (*P* = 0.016). Table 2 shows the relationship between clinicopathological data of patients with NPC and the serum levels of IGFBP-1, IGF-1, and IGFBP-1/IGF-1 ratio.

**Table 2:** Clinicpathological associations of IGF-1 and IGFBP-1 expression levels and IGF-1/IGFBP-1

Variables	Cases	IGFBP-1(ng/L)	Significance	IGF-1(ng/L)	Significance	IGFBP-1/IGF-1		Significance
	(*n*)	(Mean ± SD)	(*P*)*	(Mean ± SD)	(*P*)*	>1	≤1	(*P*)*
Gender								
Male	118	55.08 ± 41.66	0.966	88.26 ± 60.75	0.018*	38	80	0.202
Female	24	55.93 ± 40.05		135.98 ± 88.78		4	20	
Age (y)								
< 46	73	47.18 ± 36.51	0.020*	103.65 ± 69.31	0.217	16	57	0.016*
≥ 46	69	63.85 ± 44.47		89.02 ± 66.48		28	41	
Tumor size								
T1 + T2	27	47.54 ± 36.71	0.260	87.09 ± 55.78	0.437	7	20	0.528
T3 + T4	115	57.01 ± 42.18		100.71 ± 74.63		37	78	
Lymphoid nodal states								
N0-1	76	51.02 ± 41.80	0.156	96.69 ± 75.17	0.946	20	56	0.197
N2-3	66	59.99 ± 40.41		99.79 ± 67.59		24	42	
Clinical Stage								
1 + 2	15	39.11 ± 37.45	0.101	99.42 ± 58.73	0.853	3	12	0.498
3 + 4	127	57.11 ± 41.40		97.99 ± 73.02		41	86	
Local-regional relapse								
Yes	16	49.59 ± 39.68	0.537	117.07 ± 88.62	0.198	4	12	0.793
No	126	55.94 ± 41.55		95.76 ± 69.08		40	86	
Metastasis								
Yes	4	43.16 ± 41.80	0.542	58.52 ± 27.71	0.263	1	3	0.775
No	138	55.96 ± 41.20		97.42 ± 68.94		43	95	
WHO histological classification								
NKUC	131	57.05 ± 41.94	0.044*	98.25 ± 73.73	0.978	44	87	0.048*
NKDC	11	38.18 ± 25.70		96.88 ± 36.79		0	11	
EA								
≤ 1:10	53	44.34 ± 36.35	0.015*	104.31 ± 78.59	0.431	10	43	0.016*
> 1:10	89	61.64 ± 42.79		94.51 ± 67.14		34	55	
VCA								
≤ 1:40	14	57.45 ± 43.73	0.860	102.20 ± 50.26	0.736	2	12	0.263
> 1:40	128	55.39 ± 41.05		95.68 ± 70.16		42	86	

*P < 0.05, as determined by Pearson’s *Χ* ^2^ test

### Prognostic significance of serum IGF-1 and IGFBP-1 levels in NPC patients

**Fig. 5:**
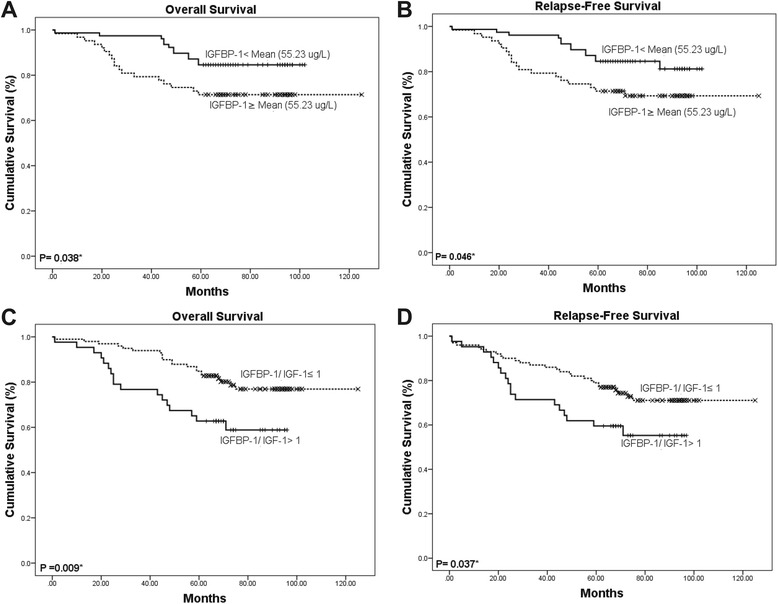
Kaplan-Meier survival analysis in patients with NPC. **a**, **b** Overall survival and Relapse-free survival curves for patients according to serum level of IGFBP-1. **c**, **d** Overall survival and relapse-free survival curves for patients according to the ratio level of IGFBP-1 to IGF-1 serum levels

Patients were classified into two groups according to their mean IGFBP-1 level (<55.23 μg/L vs. ≥ 55.23 μg/L) and IGFBP-1/IGF-1 ratio (1:1), respectively. The OS and RFS of patients with NPC were plotted using the Kaplan-Meier method, and a log-rank test was employed to evaluate the prognostic significance of IGFBP-1 levels and IGFBP-1/IGF-1ratio. The group with lower IGFBP-1 levels (<55.23 μg/L) displayed a significantly better 5-year survival rate compared to the group with higher IGFBP-1 levels (≥55.23 μg/L; Fig. 5a-b). The cumulative 5-year survival rate in the lower IGFBP-1 group was 87.6% compared to the 71.4% rate observed in the higher IGFBP-1 group (*P* = 0.038). Kaplan-Meier survival analysis revealed that higher serum IGFBP-1 levels significantly correlated with adverse RFS (*P* = 0.046) and OS (*P* = 0.038). Serum IGF-1 levels did not correlate with RFS or OS (data not shown). The IGFBP-1/IGF-1 ratio was shown to significant correlate with RFS and OS (*P* = 0.037, *P* = 0.009, respectively).

To determine whether IGFBP-1 levels or the IGFBP-1/IGF-1 ratio could potentially be used as an independent prognostic factor for NPC patient outcome, we performed a multivariate analysis for survival using a multivariate Cox regression model with respect to OS and RFS (Table 3). Gender (*P* = 0.042; *P* = 0.022), lymphoid nodal states (*P* = 0.050; *P* = 0.023) and local-regional relapse (*P* = 0.047; *P* < 0.001), metastasis (*P* = 0.006; *P* = 0.028), and the IGFBP-1/IGF-1 ratio (*P* = 0.035; *P* = 0.044) significantly correlated with OS and RFS. Thus, our findings indicate that the ratio of IGFBP-1/IGF-1 represents an independent prognostic factor for NPC outcome.

**Table 3:** Multivariate Cox regression analysis for OS of 142 patients with NPC

Characteristic	Overall Survival			Relapse-Free Survival		
	HR	95% CI	*P* value	HR	95% CI	*P* value
Gender						
Male vs. Female	0.225	0.053–0.950	0.042*	0.247	0.075–0.819	0.022*
Age (y)						
< 46 vs. ≥46	1.389	0.673–2.867	0.374	2.099	1.027–4.293	0.042*
Tumor size						
T1 + T2 vs. T3 + T4	0.873	0.292–2.611	0.809	0.972	0.325–2.905	0.959
Lymphoid nodal states						
N0-1 vs. N2-3	2.312	0.969–5.519	0.050*	2.662	1.146–6.187	0.023*
Clinical Stage						
1 + 2 vs. 3 + 4	0.798	0.327–1.945	0.619	0.856	0.284–2.579	0.783
Local-regional relapse						
Yes vs. NO	2.296	1.013–5.204	0.047*	23.877	9.554–59.668	0.000*
Metastasis						
Yes vs. No	9.085	1.871–44.128	0.006*	5.045	1.195–21.288	0.028*
WHO histological classification						
NKUC vs. NKDC	0.680	0.148–3.121	0.620	0.580	0.129–2.609	0.477
EA						
≤ 1:10 vs. >1:10	0.568	0.234–1.381	0.212	0.679	0.275–1.677	0.401
VCA						
≤ 1:40 vs. >1:40	0.613	0.207–1.816	0.377	0.376	0.131–1.084	0.070
IGFBP-1						
Low vs. High	0.802	0.330–1.947	0.626	0.520	0.213–1.267	0.150
IGF-1						
Low vs. High	1.649	0.612–4.443	0.323	1.450	0.602–3.490	0.407
IGFBP-1/IGF-1						
> 1 vs. ≤1	0.298	0.096–0.920	0.035*	0.334	0.115–0.969	0.044*

## Discussion

Here, our study showed that patients with NPC display significantly higher serum levels of IGFBP-1 and significantly lower serum levels of IGF-1 compared to healthy control individuals. Moreover, we observed that higher serum IGFBP-1 levels and an IGFBP-1/IGF-1 ratio significantly correlated with decreased overall survival.

In this study, IGF-1 was more weakly expressed in the majority of nasopharyngeal tumour and normal epithelial cell lines, which is in line with previous studies showing that IGF-1 expression is lower in EBV-negtive NPC cell lines [15]. In addition, IGF-1 expression was also weaker in NPC tumour tissues compared to adjacent normal tissues. Together, these results are consistent with the lower IGF-1 serum levels observed in NPC patients compared to the healthy controls in our study.

Our result that IGF-1 is reduced in NPC patients contradicts those of other studies showing that serum IGF-1 levels are elevated in cancer patients. High serum concentrations of IGF-1 have been associated with an increased risk of breast, prostate, colorectal and HCC [16, 17]. However, expression of IGF-1 appears to be inconsistent across different types of tumours. For example, IGF-1 protein expression has not been detected in the serum of patients with adrenocortical carcinoma [18], and IGF-1 mRNA levels are weak or absent in oesophageal squamous cell carcinoma cell lines [19]. Moreover, no alterations in IGF-1 mRNA levels are found in head and neck squamous cell carcinoma (HNSCC) [20]. However, in line with our findings, lower serum levels of IGF-1 have been reported in oral cancer patients compared to healthy controls [14]. In addition, reduced IGF-1 serum levels have been reported in epithelial ovarian cancer [21] and lower IGF-1 mRNA levels have been observed in HCC compared to corresponding non-malignant liver tissues [22]. Thus, it is possible that serum levels of IGF-1 are dependent on the type of tumour, as well as the local release ratio of IGF-1.

Although IGF-1 functions as an epithelial cell mitogen and has been in implicated in cancer development [23], increased IGF-1 levels have not been associated with tumour malignancy in some models. For example, hepatic IGF-I-deficient mice with reduced circulating IGF-I levels showed a reduced incidence of colon and breast tumorigenesis [24, 25]; however, this effect was not observed in models of prostate cancer or osteosarcoma [26, 27]. Furthermore, transgenic mice with modestly increased serum IGF-I levels did not show an increased onset or progression of breast tumorigenesis [28]. These results contradict a previous study showing that higher IGF-1 levels were associated with cancer mortality [16], but are consistent with our NPC study, suggesting that IGF-1 may play an important role in the development and progression of specific tumour types, such as NPC.

IGFBP-1 is a hepatocyte-derived secreted protein that undergoes various phosphorylation events and localizes to the nucleus and/or cytoplasm in hepatocellular carcinoma [29, 30]. In our study, however, IGFBP-1 was mainly observed within the nucleus of cancer cells and in the cytoplasm of normal cells, which is consistent with previous work showing nuclear localization of IGFBP −2, −3 -5, and −6 in tumour cells [29, 30].

By binding and sequestering IGF-1, IGFBP-1 antagonized IGF-1 which could stimulate the proliferation of cancer cells [31]. However, accumulating evidence supports the idea that IGFBPs may drive cancer, rather than exert tumour suppressive functions in some tumour types. For example, IGFBP-2 induction has been shown to activate cell invasion, and increased levels of IGFBP-2 have been reported in ovarian tumour tissues and serum [21], as well as increased IGFBP-3 mRNA expression in HNSCC compared to healthy tissue [20]. In our study, an increased serum level of IGFBP-1 was detected in patients with NPC, which is consistent with findings observed for oral cancer [8], which suggests that IGFBP-1 may play a cancer-promoting role in NPC rather than a tumour-suppressing role.

Although clear evidence that tumour growth or migration is stimulated by IGFBP-1 is still lacking, IGFBP-1 has also been repored to have IGF-independent actions, such as activating α5β1 integrin [32]. Preclinical *in vivo* work has shown that deletion of IGFBP-I in the c-Myc transgenic mouse model resulted in decreased proliferation of prostatic tissue but had no effect on the development of prostate cancer [33]. Recently, one study showed that high expression levels of MMP9 and IGFBPs were associated with poor prognosis in patients with breast cancer [34]. In contrast, high expression of IGFBPs was associated with a favorable prognosis in patients with breast cancer when MMP9 was expressed at low levels. Their study suggests that in the presence of high MMP9 levels, IGFBP is digested, and then IGF is released and activates IGF signaling pathways that promote tumorigenesis in breast cancer. Because studies indicate that high levels of MMP9 expression are observed in most NPC tissues [35], the interaction between MMP9 and IGFBP-1 in NPC tumours may contribute to NPC patients with higher levels of IGFBP-1 and an unfavourable survival. While this represents an attractive hypothesis, the underlying mechanism remains unclear and should be investigated by further studies.

In addition, the balance between IGFs and IGFBPs may represent an important factor in tumour progression [23]. While higher levels of IGF-I and IGF-I/IGFBP-3 ratio are associated with an increased risk of death from breast cancer and CRC [36, 37], alternative findings have been observed in other studies. For example, elevated IGFBP-2 and reduced IGF-1 levels or high levels of an IGFBP-2/IGF-1 ratio were shown to stratify an ovarian cancer patient subgroup with poor prognosis [21]. Similar to this result, our study showed that a higher IGFBP-1/IGF-1 ratio predicts NPC patients with unfavourable survival. Our data indicate that IGFBP-1 may play a more important role in NPC progression than IGF-1.

Iwakiri et al. reported that EBV infections induce the expression of IGF-1 mRNA and support the growth of NPC-derived cell lines [15]. EBV latent membrane protein 1 (LMP1) selectively activates IGF1R by increasing IGF-1 expression and alters the phosphorylation of IGF1R, but not the expression [38]. However, increasing levels of IGF-1 have not been associated with EA or VCA-titre levels in NPC patients in our data, although the EBV-positive C666 cell lines were characterized by higher IGF-1 expression, which is in line with Iwakiri’s findings regarding EBV-positive NPC cell lines [15], suggesting that the circulating levels of IGF-1 are inconsistent with IGF-1 expression after EBV infection *in vitro*. However, the increasing level of IGFBP-1 was associated with EA-positive, but not VCA-positive NPC sera, suggests that the association of EBV infection and the role of IGFs in NPC require further investigation.

Taken together, our study shows that higher serum IGFBP-1 levels and IGFBP-1/IGF-1 ratio correlate significantly with decreased overall survival in NPC patients. Further validation of these results is needed to determine the potential usefulness of these biomarkers for risk assessment.

## Conclusions

Our data reveal that IGFBP-1 expression is upregulated in NPC cell lines and NPC tumour tissues and that IGFBP-1 serum levels are elevated in NPC patients. In addition, we showed that IGF-1 is more weakly expressed in NPC cell lines and tumour tissues and that decreasing serum levels of IGF-1 are observed in NPC patients. Furthermore, we observed that elevated serum levels of IGFBP-1 were significantly associated with shorter OS and RFS in NPC patients. Moreover, higher levels of IGFBP-1 and lower levels of IGF-1 were shown to predict worse outcome in NPC patients, suggesting that the ratio of serum IGFBP-1/IGF-1 represents a potential biomarker for NPC patient prognosis. These findings also highlight the more complex biological activities of IGFBP-1 and IGF-1 and reinforce the need to further clarify the role of the IGF system in NPC.
